# Analyzing and identifying predictable time range for stress prediction based on chaos theory and deep learning

**DOI:** 10.1007/s13755-024-00280-z

**Published:** 2024-03-06

**Authors:** Ningyun Li, Huijun Zhang, Ling Feng, Yang Ding, Haichuan Li

**Affiliations:** 1 Department of Computer Science and Technology, Tsinghua University, Beijing, 100084 China; 2 China Huaneng Clean Energy Research Institute, Beijing, 102209 China; 3 North Automatic Control Technology Institute, Taiyuan, 030006 Shanxi China

**Keywords:** Stress prediction, Chaos theory, Deep neural networks, Predictable time range

## Abstract

**Propose:**

Stress is a common problem globally. Prediction of stress in advance could help people take effective measures to manage stress before bad consequences occur. Considering the chaotic features of human psychological states, in this study, we integrate deep learning and chaos theory to address the stress prediction problem.

**Methods:**

Based on chaos theory, we embed one’s seemingly disordered stress sequence into a high dimensional phase space so as to reveal the underlying dynamics and patterns of the stress system, and meanwhile are able to identify the stress predictable time range. We then conduct deep learning with a two-layer (dimension and temporal) attention mechanism to simulate the nonlinear state of the embedded stress sequence for stress prediction.

**Results:**

We validate the effectiveness of the proposed method on the public available Tesserae dataset. The experimental results show that the proposed method outperforms the pure deep learning method and Chaos method in both 2-label and 3-label stress prediction.

**Conclusion:**

Integrating deep learning and chaos theory for stress prediction is effective, and can improve the prediction accuracy over 2% and 8% more than those of the deep learning and the Chaos method respectively. Implications and further possible improvements are also discussed at the end of the paper.

## Introduction

Stress is a universal experience that affects humans in different ways. It is the body’s natural response to challenging or threatening situations that require adaptation or coping. A wide diversity of brain areas collectively sense stressful stimuli, interpret them as real or potential threats, and activate the Sympathetic-AdrenoMedullar (SAM) axis and the Hypothalamus–Pituitary–Adrenal (HPA) axis, the two major components involved in the physiological stress response [1]. With such complex stress networks, we are able to adapt to the dynamic and challenging environments, facing various life events every now and then. However, when stressful stimuli are chronic and severe, our stress system will become emotionally exhausted and overwhelmed, increasing greater risk for depression, heart disease, infectious diseases, etc. [2]. To prevent stress from detrimentally affecting health and well-being, it is quite important to aid individuals to know their stressful states, thereby enabling them to effectively manage the stress before bad consequences happen.

Psychological studies have suggested that *chaos theory* could offer a new paradigm for understanding human psychological phenomena and dynamic processes [3–8]. Chaos theory, proposed by Poincare [9] in the 1880s, aims to study highly complex and nonlinear dynamic systems that are sensitive to small changes in initial conditions. According to chaos theory, human mental state is not random or unpredictable, but rather follows some patterns that can be described by mathematical equations. These patterns are highly sensitive to initial conditions, which means that small differences in the initial state can lead to big differences in the final outcome. This property is known as the butterfly effect, implying that short-term behavior of the system is predictable, but not the long-term behavior. With chaos theory, we could elaborate and reconstruct the seemingly disordered stress sequence in a low-dimensional phase space into a high-dimensional phase space. Upon the reconstructed phase space, which encapsulates inherent patterns underlying the complex stress system, predictions of future stress and predictable time range could be well supported. Despite chaos theory demonstrating a strong vitality in human science research, it needs to model the complex system with a nonlinear equation. In practice, it is often difficult to determine accurate parameters and equations from the given stress sequence. Many times, we have to make predictions through approximation calculations, which will lead to false accumulation and affect the final prediction performance.

Presently, the availability of huge datasets for training and increase in computational power have made *deep learning* with Recurrent Neural Networks (RNN)-LSTM [10] a powerful tool for simulating the state of complex non-linear systems. These data-driven deep neural networks possess powerful global relation modeling capabilities, and can approximate any continuously differentiable function with sufficient training data. They can adapt to different scenarios by adjusting parameters and weights according to the feedback and evaluation to make prediction. Despite deep neural network models having achieved great success in algorithm design and practical applications, they rely on large-scale high-quality and representative data to train effectively and avoid overfitting or underfitting. When the distributions of training and testing data differ, the prediction performance of the model will degrade greatly. As real-world data often comes from dynamic open environments rather than static closed environments, it is difficult to guarantee the same data distribution in such scenarios. In other words, the distribution of data is not static. Compared with the distribution of training data, the distribution of testing data often exhibits unknown and difficult to observe behaviors, and this data shift constitutes a big challenge to deep learning methods. Another critical problem with deep learning networks is that they lack prior knowledge that can help them understand complex systems and generalize better.

The aim of this study is to investigate the validity of combining deep neural networks and chaos theory for stress prediction. Under the guidance of chaos theory, we enrich the modeling capability of deep neural networks by embedding inherent patterns underlying the complex chaotic stress system, and projecting one’s seemingly disordered low-dimensional stress sequence into a high-dimensional phase space. On the reconstructed phase space, people’s stress dynamics can be defined as a nonlinear equation, which can be better predicted and generalized. Also, with chaos theory, we are able to analyze and identify the predictable time range of chaotic stress systems, which is the maximum time interval for which reliable predictions can be made. According to chaos theory, the valid time range depends on the degree of sensitivity, complexity, and unpredictability of stress systems.

On the other hand, employing deep neural networks to learn from the data to simulate the non-linear chaotic stress system can hopefully overcome the difficulty in obtaining accurate parameters of the non-linear equations in the traditional chaos-based prediction methods.

In summary, the study makes the following contributions.

- We integrate deep learning and chaos theory to address the stress prediction problem, where predictable time ranges and future stress levels are to be explored. A two-layer (dimension and temporal) attention mechanism is particularly designed to let the deep learner focus on the influential dimensions and time points in the reconstructed stress phase space based on chaos theory.
- We investigate the validity of combining deep learning and chaos theory in terms of prediction performance and the generalization ability of the proposed method. The experimental results on the Tesserae dataset show that (1) it could achieve 74.41% and 69.23% accuracy in 2-label (unstressed/stressed) and 3-label (unstressed/weak stressed/heavy stressed) stress prediction, outperforming the deep learning method and the Chaos method; (2) the designed two-layer attention mechanism could help improve the prediction accuracy by more than 2.22% and 4.25% in 2-label and 3-label stress prediction; (3) the proposed method also performed the best on another Studentlife dataset among the three methods, about 1.12% and 2.39% higher than those of the deep learning and the Chaos method in 2-label stress prediction accuracy, and 2.95% and 5.1% higher in 3-label stress prediction accuracy.

## Related work

### Stress prediction based on deep learning

Traditional stress prediction methods are mainly based on machine learning techniques such as Support Vector Machine (SVM), Logistic Regression (LR), Random Forest, Naive Bayes, Decision Tree, etc. [11, 12].

The recent dramatic improvement in the field of deep learning has shifted the focus towards deep architectures such as Recurrent Neural Network (RNN), Long Short-Term Memory (LSTM), Gated Recurrent Unit network (GRU), etc. to learn complex non-linear sequential patterns of stress from a large collection of data. For example, [13, 14] used LSTM to predict next day’s stress given student’s previous seven days’ multi-model data including daily behavioral survey, physiology data, mobile phone usage, and mobility data. Experimental results showed that LSTM could achieve better performance than the traditional SVM and LR based methods. [15] verified that LSTM could efficiently predict human mental stress given a sequence of sensory data collected from mobile phones. [16] further presented the transfer learning based on LSTM for user adaption to provide more accurate prediction for new users. [17, 18] combined LSTM with a locally connected Multi Layer Perceptron (LC-MLP) layer to automatically extract features from sensor data for stress prediction. [19] trained MLP, LSTM, and GRU in a participant-independent fashion to evaluate the effectiveness of stress prediction based on wearable sensors in a natural environment. [20] designed a RNN and ensemble learning based stress prediction method, demonstrating that multiple predictive models can be used to yield more accurate stress prediction performance.

In addition to recurrent networks, other neural networks such as Convolutional Neural Network (CNN) and memory networks were applied to handle different types of data for stress prediction. For instance, [21] applied CNN to users’ acceleration, blood volume pulse, electrodermal activity for stress prediction, and [22] employed a neural network with one hidden layer for stress prediction, and proved that the deep learning model can be used to predict stress based on heart rate, skin conductance, sitting position, g-forces sensors values. [23] applied deep learning architectures such as AlexNet, ResNet, and DenseNet on offline handwritten signatures for stress prediction. [24] used Gaussian sampling for user-specific information, domain rules for low-frequency data, and CNN-LSTM for high-frequency data to process different types of features. [25] performed gravitational search algorithm (GSA) based feature selection with deep belief network (DBN) model (GSAFS-DBN) to predict stress among working employees. The proposed solution could select optimal features and classification process, outperforming other baseline models. [26] proposed a deep joint memory network for modeling the dynamics of users’ emotions incurred by the extracted events from their social media posts, and learned users’ personality traits based on linguistic words and a fuzzy neural network for stress prediction.

To address the issue of missing and sparse data, [27] designed Data Completion with Diurnal Regularizers to recover incomplete sensor data from wearable devices, and then built a Temporally Hierarchical Attention Network to encode daily and weekly behaviors hierarchically for stress prediction. Through multi-task learning, personalized stress prediction could be well supported [28–30].

### Chaos theory and its applications to health psychology

The idea of dynamical chaos was first glimpsed by Poincaré [9] in the 1880s. In the early 1960s, meteorologist Lorenz recognized the chaotic behavior that small differences in a dynamic system like the atmosphere could trigger vast and often unsuspected results in weather forecasts, and developed chaos theory to understand and model non-linear dynamic systems that are highly sensitive to initial conditions, seemingly random but are actually deterministic [31]. More comprehensive discussions of chaos theory and chaotic analysis techniques can be found in [32–35].

In the context of health psychology, chaos theory has been recognized as a powerful tool to investigate complex psychological phenomena [6, 8]. [36] showed that behavior problems, causal variables, and causal relationships have complex nonlinear relationships in psychological assessment, and using chaos phase space functions to simulate their nonlinear dynamic changes could better help psychological assessment. With chaos theory, [37] uncovered the clinical evolution of patients with affective instability. [3] adopted chaos theory to analyze emotion dysregulation and emotional vulnerability in adults. It utilized the Lyapunov function to assess subjects’ inconsistency in assessing their own mechanisms of emotional dysregulation under the butterfly effect. The experimental results demonstrated that the presence of initial instability to weak disturbances may herald future abnormal emotional functioning. [38] conducted a case study through a depression patient to present the butterfly effect in human mental states, showing that nonlinear dynamics and chaos theory could help understand human behaviors. [39] studied the dynamical structure of bipolar patients, and found that the self-evaluation emotional scores of the bipolar disorder patients can be described as a low-dimensional chaotic process. [5] confirmed that this kind of low-dimensional chaotic process also exists in unipolar depression.

## Methodology

Let *S* = $(s_1,s_2,\ldots , s_n)$ be a sequence of user’s daily stress levels, where $s_i$ denotes the stress level at the *i*-th day ($1 \le i \le n$), and $DOM(s_i)=\{0,1,2,3,4,5\}$, corresponding to stress level *unknown*, *no stress at all*, *very little stress*, *some stress*, *a lot of stress*, and *a great deal of stress*. Our stress prediction task aims to address the following two subtasks:

1. identify predictable time range *m*, within which reliable predictions can be made;
2. predict daily stress levels $(s_{n+1},s_{n+2},\ldots , s_{n+m})$ on the next *m* days.

We address the first subtask by conducting chaos analysis on the given stress sequence, and then leverage chaos theory and deep learning to address the second subtask. Before the discussion, let’s briefly review chaos theory and deep learning.

### A brief introduction to chaos theory and deep learning

#### Chaos theory

Chaos theory is a branch of mathematics and science that studies the behavior of nonlinear dynamical systems [35]. In order to study the chaotic characteristics of nonlinear systems, it is important to firstly recover the dynamics of a system from a single observed variable [40], known as *phase space reconstruction*. Phase space reconstruction can be achieved based on Takens’ theorem [41], which guarantees that the reconstructed phase space is topologically equivalent to the original one as long as we choose a sufficiently large embedding dimension and an appropriate time delay. [42–45] provided methods for choosing the appropriate embedding dimension, and [46] presented the way for choosing an appropriate time delay.

In the reconstructed phase space, we can explore the complexity of the nonlinear system, and estimate the amount of chaos in the nonlinear system through *the largest Lyapunov exponent*. Here, the largest Lyapunov exponent is a quantity that characterizes the rate of separation of infinitesimally close trajectories in the dynamical system. It is a measure of the sensitivity to initial conditions or the predictability of the system [33]. Usually, if the value of the largest Lyapunov exponent is positive, the presence of chaos can be determined.

The time range for accurate prediction of a chaotic system can then be estimated as a function of the largest Lyapunov exponent [32]. [47–51] provided methods for calculating the largest Lyapunov exponent based on equations and definitions. A further improvement of the method was made in [52].

#### Deep learning

Deep learning is a sub-branch of machine learning that uses artificial neural networks with multiple layers to learn from data. It can handle unstructured data such as images, texts, audio, and video, and learn features from the data automatically without human intervention. The history of deep learning can be traced back to the 1940s, when Warren McCulloch and Walter Pitts explored the idea of artificial neural networks and proposed the McCulloch–Pitts neuron [53]. In 1986, Geoffrey Hinton popularized the back-propagation algorithm for training multi-layer neural networks [54], which caused another upsurge in neural networks.

Afterwards, a series of neural networks such as Convolutional Neural Networks (CNN) [55] and Recurrent Neural Networks (RNN) [56] were subsequently proposed. CNN [55] used convolutional layers to extract features from images, which was widely used for image classification, object detection, face recognition, etc. RNN [56] employed recurrent layers to process sequential data such as text, speech, and video. They can capture dependencies and context in the data. Some variants of RNN like Long Short-Term Memory Network (LSTM) [57] and Gated Recurrent Unit (GRU) [58] have been developed to overcome the problem of vanishing gradients, and learn dependencies in sequential data, which were widely used for natural language processing, speech recognition, etc.

### Identification of predictable time range *m* (subtask 1)

#### Phase space reconstruction

We project the user’s original stress sequence $S=(s_1,s_2,\ldots , s_n)$ into a high-dimensional phase space:

1 $$\begin{aligned} X(\tau ,d) = (X_1,X_2,\ldots ,X_{n-(d-1)\tau }) \end{aligned}$$

where $X_k=(s_k, s_{k+\tau },\ldots ,s_{k+(d-1)\tau }) \in {\mathbb {R}}^{d}$, $\tau$ is the time delay determining the distance between the two successive points, $s_k$ and $s_{k+\tau }$, in the phase space, and *d* is the embedding dimension of the phase space for $k=1,2,\ldots ,n-(d-1)\tau$.

The setting of $\tau$ is to maximize the knowledge about $s_{k+\tau }$ from $s_k$ and minimize the redundancy between $s_{k+\tau }$ and $s_k$. [46] presented a way to set $\tau$ by computing and minimizing the mutual information *M* between $(s_1,s_2,\ldots ,s_{n-\tau })$ and $(s_{1+\tau },s_{2+\tau },\ldots ,$ $s_{n-\tau +\tau })$. The less the mutual information, the less dependence between the two variables.

2 $$ \begin{aligned} M&= \sum \limits_{i=1}^{n-\tau }~\sum _{j=1+\tau }^{n}P(s_i,s_j) \cdot log_2 ~P(s_i,s_j) \\&\quad - \sum  \limits_{i=1}^{n-\tau }P(s_i) \cdot log_2 ~P(s_i) - \sum \limits_{j=1+\tau }^{n}P(s_j) \cdot log_2 ~P(s_j) \end{aligned} $$

where $P(s_i)$ and $P(s_j)$ are the probabilities of $s_i$ and $s_j$ in $(s_1,s_2,\ldots ,s_{n-\tau })$ and $(s_{1+\tau },s_{2+\tau },\ldots ,$ $s_{n-\tau +\tau })$, respectively, and $P(s_i,s_j)$ is the joint probability distribution of $s_i$ and $s_j$. When *M* drops to the local minimum value for the first time, the corresponding value of $\tau$ is the optimal delay time $\tau$.

We adopted the method proposed in [43] to determine the minimum embedding dimension value *d*. The basic idea is that, since the chaotic sequence is the projection of the high-dimensional chaotic system in the one-dimensional space, during the projection, some non-adjacent points in the high-dimensional space will become adjacent when projected into one-dimensional space, forming false nearest neighbors. When the embedding dimension gradually increases, the false nearest neighbors gradually disappear. When the number of false nearest neighbors drops to 0, a suitable embedding dimension value is obtained. Let $X_k(d)=(s_k, s_{k+\tau },\ldots ,s_{k+(d-1)\tau })$ be a vector in the phase space $X(\tau ,d)$. In this *d*-dimensional space, each vector $X_k(d)$ has its nearest neighbor $X_{n(k,d)}(d)$, where $n(k,d)\in \{1,\ldots ,n-(d-1)\tau \}$ and $n(k,d)\ne k$. The distance between $X_k(d)$ and $X_{n(k,d)}(d)$, denoted as $R_k(d)$, can be computed as:

3 $$\begin{aligned} \begin{aligned} R_k(d)&= ~ ||~ X_k(d) - X_{n(k,d)}(d) ~|| \\&= ~ \sqrt{\sum \limits _{0\le j \le d-1}^{}|s_{k+j\tau }-s_{n(k,d)+j\tau }|^2} \end{aligned} \end{aligned}$$

where $||\cdot ||$ denotes the Euclidean norm.

When the dimension of the phase space increases from *d* to ($d+$ 1), the distance between the two points will change to:

4 $$\begin{aligned} R_k(d+1) = ||~X_k(d+1)-X_{n(k,d)}(d+1)~|| \end{aligned}$$

Whenever $R_k(d+1)$ is much larger than $R_k(d)$, the two nearest neighbors can be regarded as false nearest neighbors. The distance ratio *r*(*k*, *d*) of the nearest neighbors in the *d*-dimensional space and ($d+1$)-dimensional space can be computed as:

5 $$\begin{aligned} \begin{aligned} r(k,d)&= \frac{R_k(d+1)}{R_k(d)}\\&=\frac{||X_k(d+1)-X_{n(k,d)}(d+1)||}{||X_k(d)-X_{n(k,d)}(d)||} \end{aligned} \end{aligned}$$

When *r*(*k*, *d*) is larger than a threshold, $X_k(d)$ and $X_{n(k,d)}(d)$ become false nearest neighbors. We calculate the average distance ratio of all nearest neighbor pairs in the *d*-dimensional space and ($d+1$)-dimensional space by:

6 $$\begin{aligned} E(d) = \frac{1}{n-(d-1)\tau }\sum \limits _{k=1}^{n-(d-1)\tau }r(k,d) \end{aligned}$$

When the false nearest neighbors gradually decrease along with the increase of *d*, the changes of *r*(*k*, *d*) and of *E*(*d*) tend to be stable. To measure the variation from *d* to ($d+1$), we define:

7 $$\begin{aligned} EV(d) = \frac{E(d+1)}{E(d)} \end{aligned}$$

When *EV*(*d*) stops changing at a certain value $d_0$, it means that the change of *E*(*d*) becomes stable, and the number of false nearest neighbors tends to 0. In this case, the result $d_0$ is the minimum embedding dimension value we are looking for.

#### Largest Lyapunov exponent

The largest Lyapunov Exponent is an important factor for judging whether the sequence is chaotic. It indicates the average exponential divergence rate of adjacent trajectories in the phase space. A positive Lyapunov Exponent means that no matter how small the distance between the two trajectories in the initial state, the distance between them will increase exponentially over time. This is one of the most typical features of a chaotic system, so if the value of the largest Lyapunov Exponent is positive, the presence of chaos can be determined.

According to [32], the predictable time range of a chaotic system can be defined as the length of time before small differences in the initial state of the system begin to change exponentially, which is the reciprocal of the largest Lyapunov Exponent $l_{max}$ of the stress sequence. Therefore, the predictable time range *m* can thus be inferred as:

8 $$\begin{aligned} m = \frac{1}{l_{max}} = \frac{1}{D^{\prime}(q)} \end{aligned}$$

where $l_{max}$ is the slope of function *D*(*q*), i.e., the derivative of *D*(*q*) [52]:

9 $$\begin{aligned} D(q) = \frac{1}{n-(d-1)\tau }\sum \limits _{k=1}^{n-(d-1)\tau }ln~||X_{n(k,d)+q}-X_{k+q}|| \end{aligned}$$

Here, $X_{n(k,d)}$ is the nearest neighbor of $X_k$.

### Prediction of stress levels on the next *m* days (subtask 2)

**Fig. 1:**
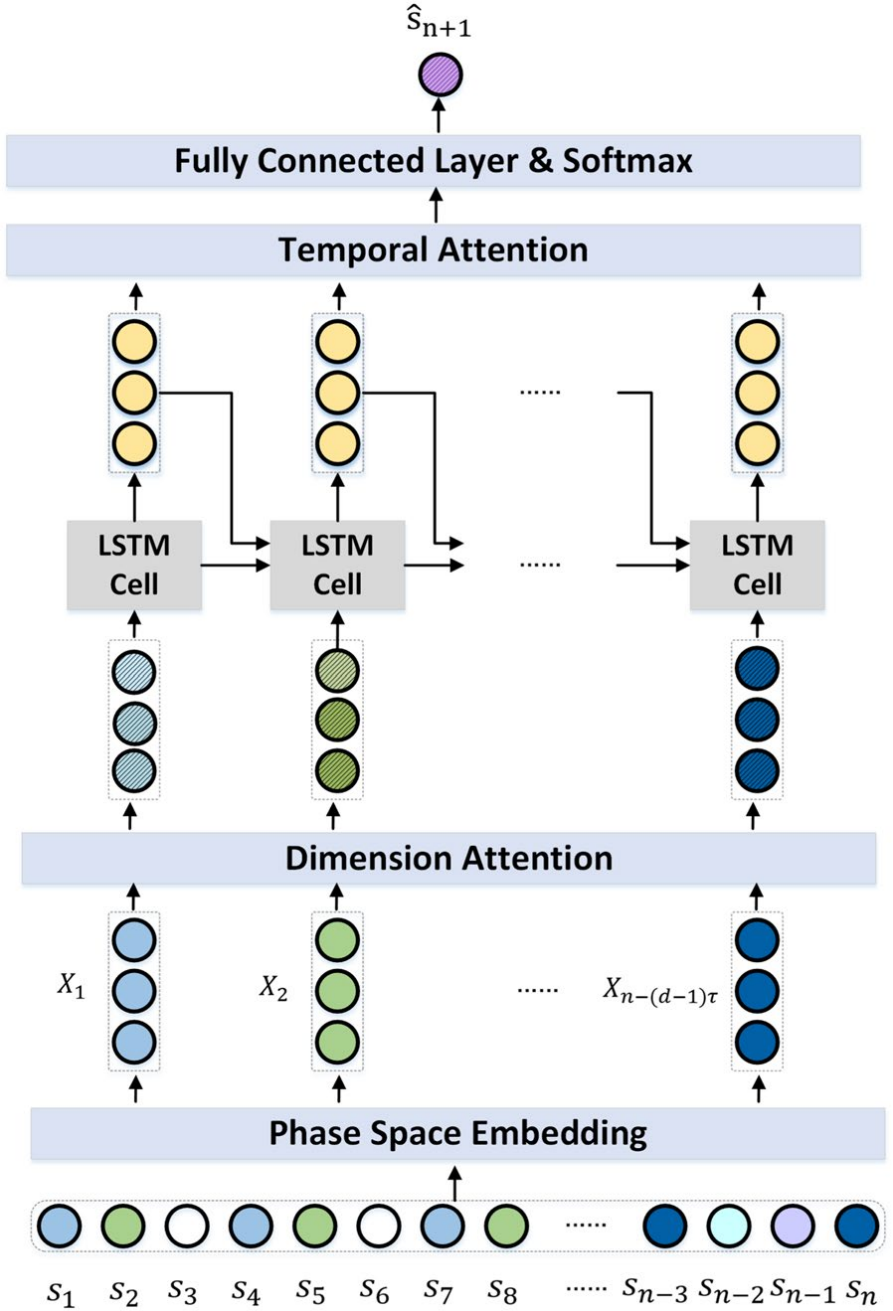
Chaos and deep learning based stress prediction framework

Figure 1 shows our chaos and deep learning based stress prediction framework. The stress sequence $S=(s_1,s_2,\ldots ,s_n)$ was firstly embedded with chaos dynamic patterns into a high *d*-dimensional phase space $X(\tau ,d)=(X_1,X_2,\ldots \,X_{n-(d-1)\tau })$, where $X_k=(s_k,s_{k+\tau },\ldots ,s_{k+(d-1)\tau })$ (for $k=1,2,\ldots ,n-(d-1)\tau$). To ensure that our model can handle variable-length input sequences in long-term prediction, we applied two ways of zero-padding in the input sequence. We added 0 before and after the input sequence respectively to make the sequence reach the longest input length (44 in this paper), and fed these two sequences into the model at the same time for training. We enforced *dimension attention* upon each *d*-dimensional vector in $X(\tau ,d)$, and then fed the dimension-attended $X_1,X_2,\ldots \,X_{n-(d-1)\tau }$ into respective LSTMs chained with *temporal attention* to learn the stress sequence representation.

*Dimension attention.* The dimension attention *DA* can be computed as:

10 $$\begin{aligned} \begin{aligned} DA&= tanh((X(\tau ,d))^T\times W_{DA}+b_{DA}) \end{aligned} \end{aligned}$$

where $DA \in {\mathbb {R}}^{d}$, and $W_{DA}\in {\mathbb {R}}^{{n-(d-1)\tau }\times 1},b_{DA} \in {\mathbb {R}}^ {1 \times 1}$ are trainable parameters.

With *dimension attention*, we can get the dimension-attended $X_1,X_2,\ldots \,X_{n-(d-1)\tau }$ denoted as $X^{\prime}(\tau ,d)$:

11 $$\begin{aligned} \begin{aligned} X^{\prime}(\tau ,d) = X(\tau ,d) \times DA \end{aligned} \end{aligned}$$

Then $X^{\prime}(\tau ,d) = (X^{\prime}_1,X^{\prime}_2,\ldots \,X^{\prime}_{n-(d-1)\tau })$ is fed into LSTM:

12 $$\begin{aligned} \begin{aligned} h_k&= LSTM(h_{k-1},{X^{\prime}_k}) \end{aligned} \end{aligned}$$

*Temporal attention.* The temporal attention *TA* can be computed as:

13 $$\begin{aligned} \begin{aligned} TA&= Softmax(H\times W_{TA}+b_{TA}) \end{aligned} \end{aligned}$$

where $H=(h_1, h_2, \ldots , h_{n-(d-1)\tau })$, $TA \in {\mathbb {R}}^{{n-(d-1)\tau }\times 1}$, and $W_{TA}\in {\mathbb {R}}^{{hidden\_size}\times 1},b_{TA} \in {\mathbb {R}}^ {1\times 1}$ are trainable parameters, $hidden\_size$ is the size of hidden state of *LSTM*, in this study, $hidden\_size=8$.

With the *temporal attention* *TA*, we can get the overall information *I* of the input stress level sequence $X(\tau ,d)$:

14 $$\begin{aligned} \begin{aligned} I = H\times TA \end{aligned} \end{aligned}$$

Through a final fully connected layer and Softmax, prediction of stress level on the next ($n+$ 1)-th day could be made.

15 $$\begin{aligned} \begin{aligned} {\hat{s}}_{n+1} = Softmax(I\times W_s + b_s) \end{aligned} \end{aligned}$$

where ${\hat{s}}_{n+1}$ represents the possibility of the user under different stress levels, $W_s \in {\mathbb {R}}^{{hidden\_size}\times num\_class}$ and $b_s \in {\mathbb {R}}^{num\_class \times 1}$ are trainable parameters, $num\_class$ is number of stress levels, in this study, $num\_class$ = 2 or 3.

With the predicted stress level ${\hat{s}}_{n+1}$, we could then form a longer stress sequence $S=(s_1,s_2,\ldots ,s_n,{\hat{s}}_{n+1})$ to predict the stress level ${\hat{s}}_{n+2}$ on the ($n+$ 2)-th day. The process repeated until stress levels $({\hat{s}}_{n+1},{\hat{s}}_{n+2},\ldots , {\hat{s}}_{n+m})$ on the next *m* days were predicted.

## Performance study

### Experimental setup

#### Dataset

A set of experiments were conducted on the publicly available Tesserae dataset [59], which enrolled 757 information workers across the United States. During the 56 days after enrollment, the participants were requested to fill out the ground truth questionnaire on a daily basis, which evaluates the participants’ daily stress at five stress levels (i.e. *no stress at all*, *very little stress*, *some stress*, *a lot of stress* and *a great deal of stress*). In the study, we selected 478 participants out of the 757 enrolled participants, who completed the daily ground truth survey for over 45 days (80% of the total 56 days). Unreported stress level on a certain day was assigned a special label “*unknown*”. Table 1 details the dataset used in the experiments.

**Table 1:** Dataset used in the experiments

Total users	Gender		Age					Stress Sequence	
	Male	Female	<25	25-34	35-44	45-54	55-64	Total length	Average missing ratio
1	295	183	49	205	132	7.60% (2.66/35)	66	26	9.47% (1.80/19)

We applied fivefold cross validation to ensure every user was used for testing. Each time, 80% of the participants were for training, and the rest 20% of the participants were for testing.

#### Comparison methods

We compared our chaos and deep learning based stress prediction method with the following two methods.

- *Chaos method*, which modeled the dynamic stress system in the *d*-dimensional Euclidean space ${\mathbb {R}}^d$ through a continuous function $X_{k+1}=F(X_k)$, where $X_k, X_{k+1}$ are two vectors in the reconstructed phase space $X(\tau ,d)$. Since $F(\cdot )$ is a continuous function, we can know that if $X_{n-(d-1)\tau }$ and $X_j$ are close to each other ($1\le j<n-(d-1)\tau$), then $X_{n-(d-1)\tau +1}$ and $X_{j+1}$ will also be close to each other in the phase space, signifying the pair-wise closeness of elements (e.g., the last elements $s_{n+1}$ and $s_{j+(d-1)\tau +1}$ in $X_{n-(d-1)\tau +1}$ and $X_{j+1}$). By searching for *q* nearest neighbors of $X_{n-(d-1)\tau }$, denoted as $X_{top-n(k,d)}$, in the phase space, the method approximated the stress level $s_{n+1}$ by:
  16 $$\begin{gathered} \hat{s}_{{n + 1}} = \sum\limits_{{\begin{array}{ll} {X_{j} \in } \\ {X_{{top - n(k,d)}} } \\ \end{array} }} {\lambda (X_{j} ,X_{{n - (d - 1)\tau }} ) \cdot s_{{j + (d - 1)\tau + 1}} } \quad \hfill \\ {\text{where}}\;\lambda (X_{j} ,X_{{n - (d - 1)\tau }} ) = \frac{{e^{{ - \frac{1}{2}||X_{j} - X_{{n - (d - 1)\tau }} ||^{2} }} }}{{\sum\nolimits_{{X_{q} \in X_{{top - n(k,d)}} }} {e^{{ - \frac{1}{2}||X_{q} - X_{{n - (d - 1)\tau }} ||^{2} }} } }} \hfill \\ \end{gathered}$$
  Like ${\hat{s}}_{n+1}$, ${\hat{s}}_{n+2}, {\hat{s}}_{n+3},\ldots ,{\hat{s}}_{n+m}$ could be estimated one by one.
- *Deep learning method*, which skipped the phase space reconstruction layer and dimension attention layer (Fig. 1), and fed the original stress sequence directly into chained LSTMs with temporal attention.

#### Evaluation metrics

We measured the performance of daily stress prediction and range stress prediction (i.e., all the daily prediction for the next *m* days) through the average accuracy, precision, recall, and F1-score of all classes:

Accuracy = (TP + TN)/(TP + FP + TN + FN);

Precision = TP/(TP + FP);

Recall = TP/(TP + FN);

F1-score = (2 $\times$ Precision $\times$ Recall)/(Precision + Recall)

where TP denotes the number of true-positive, FP denotes the number of false-positive, FN denotes the number of false-negative, and TN denotes the number of true-negative. Here, the true/false refers to the prediction outcome being correct or incorrect, while positive/negative refers to the predicted label belonging to the positive or negative class.

Test users’ “*unknown*” (unreported) real daily stress levels were not counted in the above calculations.

### Experimental results

We performed the experiments on a 8-NVIDIA GeForce RTX-2080 machine. Batch size was set to 64 during the training process, and the learning rate was set to 0.001. Adam [60] was adopted as the training optimizer.

#### Predictable time range *m*

**Fig. 2:**
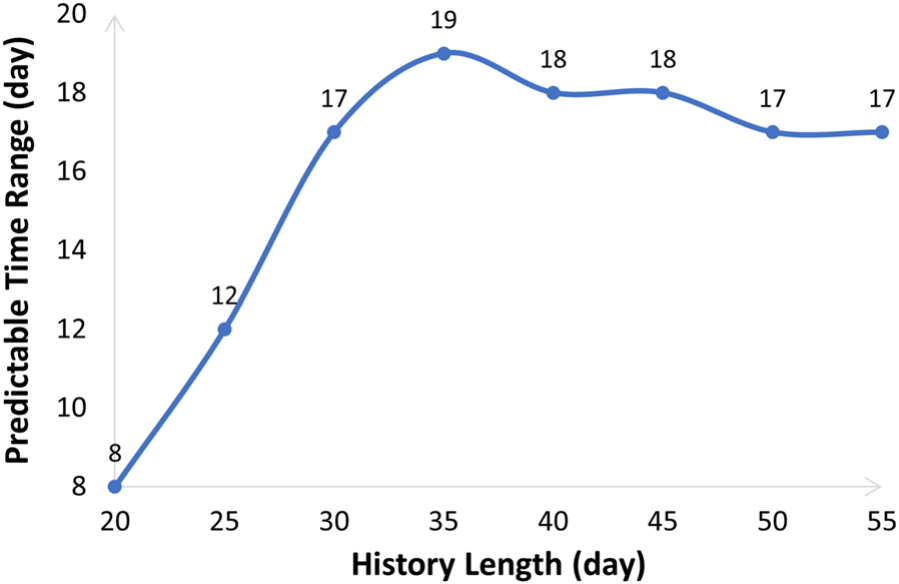
Predictable time ranges when the length of the input stress sequence varies from 20 days to 55 days

The predictable time range could be estimated based on Eq. 8 (in section “Largest Lyapunov exponent”). It varied along with the length of the input stress sequence. As shown in Fig. 2, the longer the input stress sequence, the wider the predictable time range. However, when the input sequence length comes to around 35 days, the predictable time range (19 days) stops extending. This complies with the chaotic characteristic that short-term behaviors of the chaotic system are predictable, but not long-term behaviors.

Therefore, we made three test settings in the following performance study, as shown in Table 2.

**Table 2:** Three test settings in the performance study

Test setting	Input			(Predicted) output		
	Length	Stress sequence	Average missing ratio	Length	Stress sequence	Average missing ratio
1	$n=$ 35 days	$(s_1,s_2,\ldots ,s_{35})$	7.60% (2.66/35)	$m=$ 19 days	$(s_{36},s_{37},\ldots ,s_{54})$	9.47% (1.80/19)
2	$n=$ 30 days	$(s_1,s_2,\ldots ,s_{30})$	7.51% (2.25/30)	$m=$ 17 days	$(s_{31},s_{32},\ldots ,s_{47})$	9.21% (1.57/17)
3	$n=$ 25 days	$(s_1,s_2,\ldots ,s_{25})$	7.36% (1.84/25)	$m=$ 12 days	$(s_{26},s_{27},\ldots ,s_{37})$	9.05% (1.09/12)

#### Daily and range stress prediction performance

We compared the 2/3-label daily and range stress prediction performance of the three methods under input length $n=$ 35 days and output length $m=$ 19 days. The 2-label assignment includes “unstressed” and “stressed”. The 3-label assignment includes “unstressed”, “weak stressed”, and “heavy stressed”.

Their range stress prediction behaviors for the next $m=$ 19 days are presented in Table 3. As shown, the proposed DL+Chaos method outperforms the Chaos and the DL methods for both 2-label and 3-label range stress prediction. The accuracy and F1-score of the DL+Chaos method achieve 74.41% and 71.59%, respectively, for 2-label stress prediction, about 2.28% and 1.71% more than those of the DL method, and 9.72% and 9.92% more than those of the Chaos method. For 3-label stress prediction, the accuracy and F1-score of the DL+Chaos method are 2.12% and 2.39% more than those of the DL method, and 8.38% and 3.43% more than those of the Chaos method. The results demonstrate the effectiveness of combining deep learning techniques and chaos analysis on range stress prediction. By combining chaos theory and deep learning method, we can better discover the law behind the stress sequence in the reconstructed phase space and simulate this law through deep learning to better predict stress levels.

**Table 3:** Range stress prediction performance, where input length *n* is 35 days and output stress length is 19 days

Methods	Label	Acc. (%)	Pre. (%)	Rec. (%)	F1. (%)
Chaos^a^	2-label	64.69	61.69	61.69	61.67
	3-label	60.85	43.86	42.17	42.11
DL^b^	2-label	72.13	71.25	70.12	69.88
	3-label	67.11	45.57	44.31	43.15
DL+Chaos^c^	**2-label**	**74.41**	**73.10**	**71.22**	**71.59**
	**3-label**	**69.23**	**46.20**	**46.28**	**45.54**

^a^Chaos (the Chaos method)

^b^DL (the Deep Learning method)

^c^DL+Chaos (the Deep Learning+Chaos method)

Bold values indicate the best experimental results in the experiment

Another observation we made from Table 3 is that for all three methods, the 3-label stress prediction performance is lower than the respective 2-label prediction performance. This is expected since the former is more challenging than the latter. The accuracy and F1-score of the DL+Chaos method decrease by 5.18% and 26.05%, respectively, and the accuracy and F1-score of the DL method decrease by 5.02% and 26.73%, respectively. Comparatively, the performance of the Chaos method decreases by 3.84% and 19.56%, respectively, the least among the three methods. This may be because the data distribution for 2-label prediction is about 1 (unstressed): 2 (stressed), while the data distribution for 3-label prediction is more bias, about 5 (unstressed): 10 (weak stressed): 1 (heavy stressed). Data-driven DL method and DL+Chaos method are thus more sensitive and affected by data distribution than the Chaos method.

**Fig. 3:**
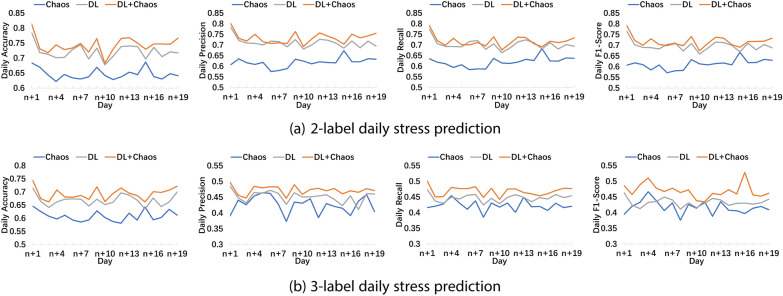
Daily stress prediction performance under input length $n=$ 35 days

Apart from range stress prediction for the next *m* days, we also investigate the daily stress prediction performance. As illustrated in Fig. 3a and b, the DL+Chaos method has the best performance among the three methods.

#### Impact of input stress sequence length on range stress prediction performance

We examined the 2/3-label range stress prediction performance of the three methods when the input length of the stress sequence varies from 35 days, 30 days, to 25 days and the corresponding output length varies from 19 days, 17 days, to 12 days (Table 2). From the results presented in Table 4, we find that the longest input length (35 days) yields the best range stress prediction performance for all three methods, despite the number of days for prediction is the most (19 days). When the input sequence length is 35 days, for 2-label stress prediction, the accuracy of the three methods are more than 0.3% and 1.76% higher than when the input length sequence is 30 and 25 days respectively, and for 3-label stress prediction, the accuracy of the three methods are respectively more than 0.22% and 1.77% higher than when the input length sequence is 30 days and 25 days. This indicates the importance of learning the historical stress sequence for future stress prediction. More historical data could help predict future stress more effectively.

**Table 4:** Range stress prediction performance when the input stress sequence length *n* varies from 35 days, 30 days, to 25 days, and the corresponding output length varies from 19 days, 17 days, to 12 days

Methods	Input	Acc. (%)	Pre. (%)	Rec. (%)	F1. (%)
(a) 2-label range stress prediction					
Chaos	35 days	64.69	61.69	61.69	61.67
	30 days	64.39	62.05	62.26	61.43
	25 days	62.93	60.63	61.20	60.45
DL	35 days	72.13	71.25	70.12	69.88
	30 days	64.51	69.54	70.55	69.05
	25 days	69.71	70.05	69.66	68.33
**DL+Chaos**	**35 days**	**74.41**	**73.10**	**71.22**	**71.59**
	30 days	72.21	70.81	70.02	70.18
	25 days	71.95	70.17	69.72	69.83
(b) 3-label range stress prediction					
Chaos	35 days	60.85	43.86	42.17	42.11
	30 days	60.63	44.27	42.03	41.24
	25 days	59.08	40.51	40.40	39.25
DL	35 days	67.11	45.57	44.31	43.15
	30 days	66.88	45.27	44.88	43.89
	25 days	65.19	44.48	44.09	43.13
**DL+Chaos**	**35 days**	**69.23**	**46.20**	**46.28**	**45.54**
	30 days	68.22	46.96	45.53	45.03
	25 days	67.74	44.58	46.17	45.01

Bold values indicate the best experimental results in the experiment

#### Impact of missing data on range stress prediction performance

According to Table 2, when input length n = 35 days, the average missing ratio of the input stress sequence is 7.60%. To investigate the impact of missing data, we increase the missing ratio of the input stress sequence to 10%, 12%, and 14% by randomly setting some stress levels in the input sequence to 0. Table 5 present the range stress prediction performance results of the three methods, respectively. We can find that in 2-label stress prediction, as the input missing ratio increases, the stress prediction accuracy and F1-score of the DL+Chaos method decrease by 2.23% and 1.17% at most, while the DL method decrease by 4.35% and 4.44% at most, and the Chaos method drop up to 7.67% and 8.56%. In 3-label stress prediction, the prediction accuracy and F1-score of the DL+Chaos method decrease by 4.85% and 0.3% at most, while the DL method decrease by 6.08% and 1.53% at most, and the Chaos method drop up to 7.42% and 2.75%. The DL+Chaos method has less performance degradation than the DL method and the Chaos method in the face of increasing missing data. This suggests that our DL+Chaos method is more stable in terms of missing data.

**Table 5:** Range stress prediction performance when the input stress sequence has different missing ratios

Missing ratio	Input	Acc. (%)	Pre. (%)	Rec. (%)	F1. (%)
(a) Chaos					
7.6	2-label	64.49	61.69	61.69	61.67
	3-label	60.85	43.86	42.17	42.11
10	2-label	62.82	60.46	60.31	60.33
	3-label	57.28	40.49	41.94	41.14
12	2-label	59.78	56.81	56.39	56.19
	3-label	55.86	40.55	40.05	41.10
14	2-label	57.02	53.74	53.07	53.11
	3-label	53.43	39.37	39.47	39.36
(b) DL					
7.6	2-label	72.13	71.25	70.12	69.88
	3-label	67.11	45.57	44.31	43.15
10	2-label	70.90	70.08	70.09	69.22
	3-label	65.15	43.02	43.27	42.04
12	2-label	68.53	66.29	67.20	66.79
	3-label	64.23	43.12	42.09	42.50
14	2-label	67.78	65.59	66.25	65.44
	3-label	61.03	42.27	41.40	41.62
(c) DL+Chaos					
7.6	2-label	74.41	73.10	71.22	71.59
	3-label	69.23	46.20	46.28	45.54
10	2-label	73.64	72.31	70.86	71.09
	3-label	67.06	45.88	46.89	45.83
12	2-label	72.18	70.45	69.71	69.82
	3-label	69.51	46.43	46.83	46.43
14	2-label	73.03	71.81	69.58	69.90
	3-label	64.38	49.06	46.72	45.25

#### Effectiveness of dimension attention and temporal attention on daily and range stress prediction performance

**Fig. 4:**
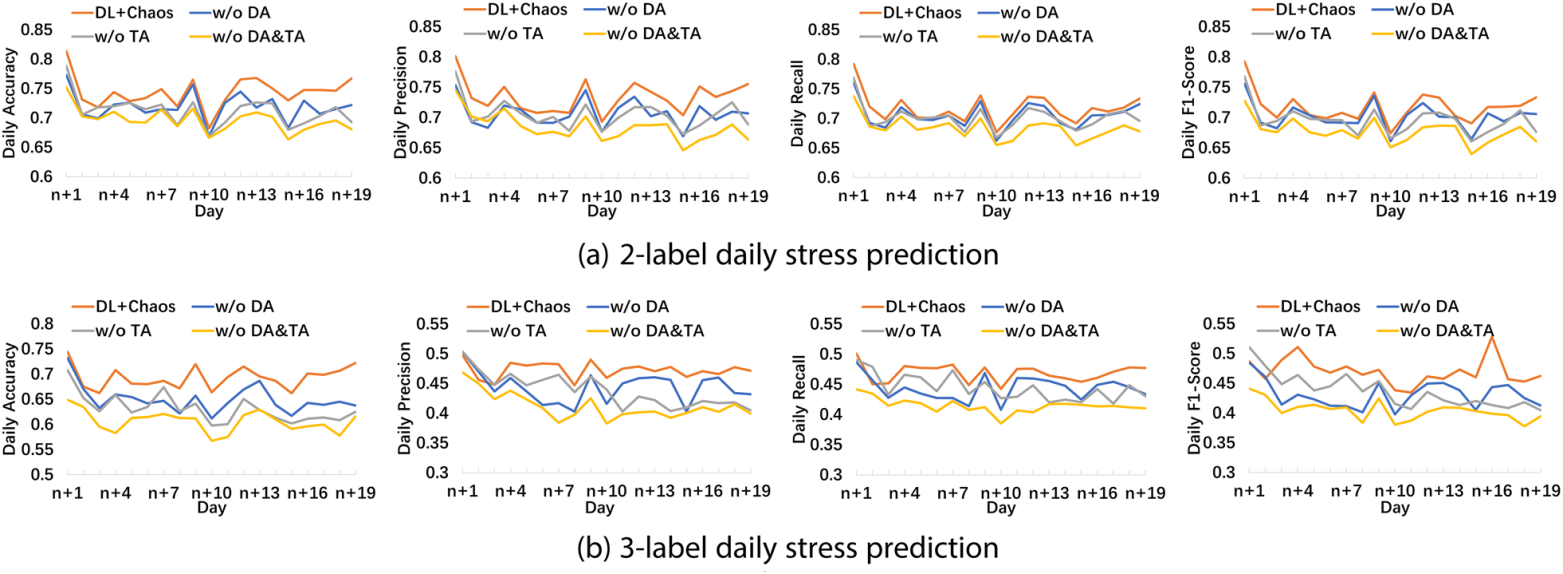
Effectiveness of dimension attention and temporal attention of the DL+Chaos method

In the study, we designed two attention mechanisms (*dimension attention* and *temporal attention*) to learn and enforce the influence of specific phase space dimensions and temporal order on stress prediction. To investigate their effectiveness, we conducted ablation studies by removing dimension attention and/or temporal attention. As shown in Fig. 4 and Table 6, removing dimension attention or temporal attention leads to a decrease in daily and range stress prediction performance, and removing both attention mechanisms brings a further drop in stress prediction performance. The accuracy can drop 2.22% (4.25%), 2.83% (6.07%) and 3.27% (7.87%) respectively when there is no dimension attention, no temporal attention, and neither for 2-label (3-label) prediction.

The results demonstrate the effectiveness of the two attention mechanisms since they could help the model focus on more important dimensions in phase space and more important time horizons.

**Table 6:** Range stress prediction performance of the DL+Chaos method when removing dimension attention and/or temporal attention

Attentions	Label	Acc. (%)	Pre. (%)	Rec. (%)	F1. (%)
w/o DA^a^	2-label	72.19	70.84	70.52	70.90
	3-label	64.98	45.36	44.48	43.49
w/o TA^b^	2-label	71.58	70.75	70.67	70.02
	3-label	63.16	45.61	45.88	44.09
w/o DA &TA^c^	2-label	71.14	69.63	69.62	69.20
	3-label	61.36	41.89	41.34	39.43
**with DA &TA**	**2-label**	**74.41**	**73.10**	**71.22**	**71.59**
	**3-label**	**69.23**	**46.20**	**46.28**	**45.54**

^a^w/o DA (without dimension attention)

^b^w/o TA (without temporal attention)

^c^w/o DA &TA (without both dimension attention and temporal attention)

Bold values indicate the best experimental results in the experiment

#### Evaluation of the generalization ability of the prediction methods

To examine the generalization ability of the three methods, we conducted the experiments on another Studentlife dataset [61]. The Studentlife dataset was collected from 48 Dartmouth college students over 10 weeks. The participants were asked to report their daily stress at five stress levels (i.e. *feeling great*, *feeling good*, *a little stressed*, *definitely stressed*, *stressed out*). In the experiments, we selected 32 users who reported their daily stress levels for more than 20 days.

**Fig. 5:**
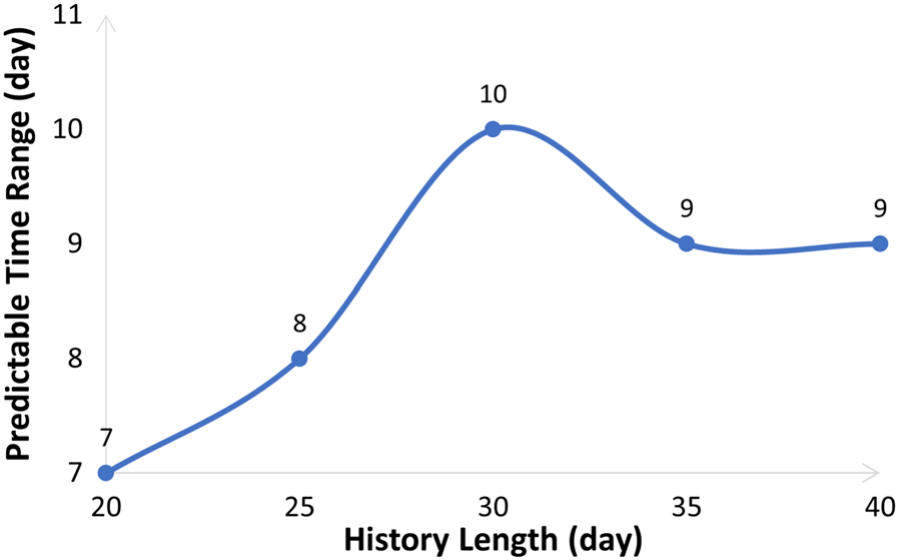
Predictable time ranges when the length of the input stress sequence varies from 20 to 40 days

**Fig. 6:**
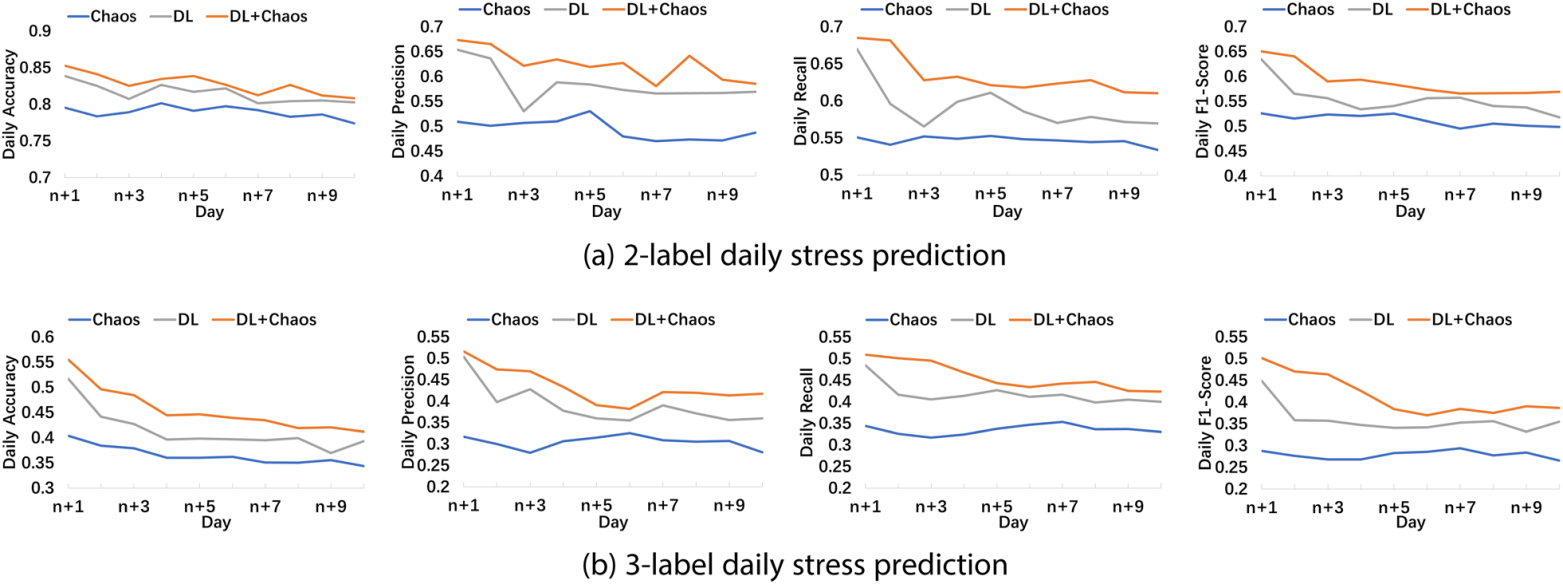
Daily stress prediction performance on the Studentlife dataset under input length n = 30 days

We calculated the predictable time ranges, as shown in Fig. 5. When the input stress sequence length is 30 days, the predictable time range reaches a maximum of 10 days. Thus, we conducted the experiments under the condition that the input sequence length is 30 days. To expand the experimental data, we regarded the user’s data every 40 days as a sample. In this way, we obtained a total of 637 samples. We applied fivefold cross validation, and ensured that different samples generated from the same user only appeared in the same fold. The experimental results of 2/3-label daily and range stress prediction on the Studentlife dataset are presented in Fig. 6 and Table 7. We can find that our proposed DL+Chaos method outperforms the Chaos and the DL method on both daily and range stress prediction. In 2-label range stress prediction, the accuracy of the three methods (the Chaos method, the DL method and the DL+Chaos method) are 79.35%, 80.62% and 81.74% respectively, the accuracy of the DL+Chaos method is 2.39% and 1.12% higher than that of the Chaos method and the DL method respectively, and the F1-Score of the DL+Chaos method is 6.86% and 4.23% higher than that of the Chaos method and the DL method respectively. In 3-label range stress prediction, the accuracy of the three methods (the Chaos method, the DL method and the DL+Chaos method) are 39.32%, 41.47% and 44.42% respectively, the accuracy of the DL+Chaos method is 5.1% and 2.95% higher than that of the Chaos method and the DL method respectively, and the F1-Score of the DL+Chaos method is 13.56% and 5.09% higher than that of the Chaos method and the DL method respectively.

**Table 7:** Range stress prediction performance on the Studentlife dataset, where input length *n* is 30 days and output stress length is 10 days

Methods	Label	Acc. (%)	Pre. (%)	Rec. (%)	F1. (%)
Chaos	2-label	79.35	47.95	52.47	50.49
	3-label	39.32	31.56	33.60	28.03
DL	2-label	80.62	61.20	56.00	53.12
	3-label	41.47	39.07	41.97	36.50
**DL+Chaos**	**2-label**	**81.74**	**63.86**	**60.47**	**57.35**
	**3-label**	**44.42**	**42.52**	**44.35**	**41.59**

Bold values indicate the best experimental results in the experiment

The results demonstrate the potential of the DL+Chaos method for more datasets compared to the pure Chaos method and the DL method.

## Discussion

The study investigates the validation of combining deep learning and chaos theory for stress prediction. The experimental results on the publicly available Tesserae dataset show that leveraging deep learning and chaos theory can improve the stress level prediction performance over a period of time, compared with the pure deep learning method and Chaos method. While the performance result is promising, a number of issues about combining deep learning and chaos theory for stress prediction still remain and deserve further study.

**Fig. 7:**
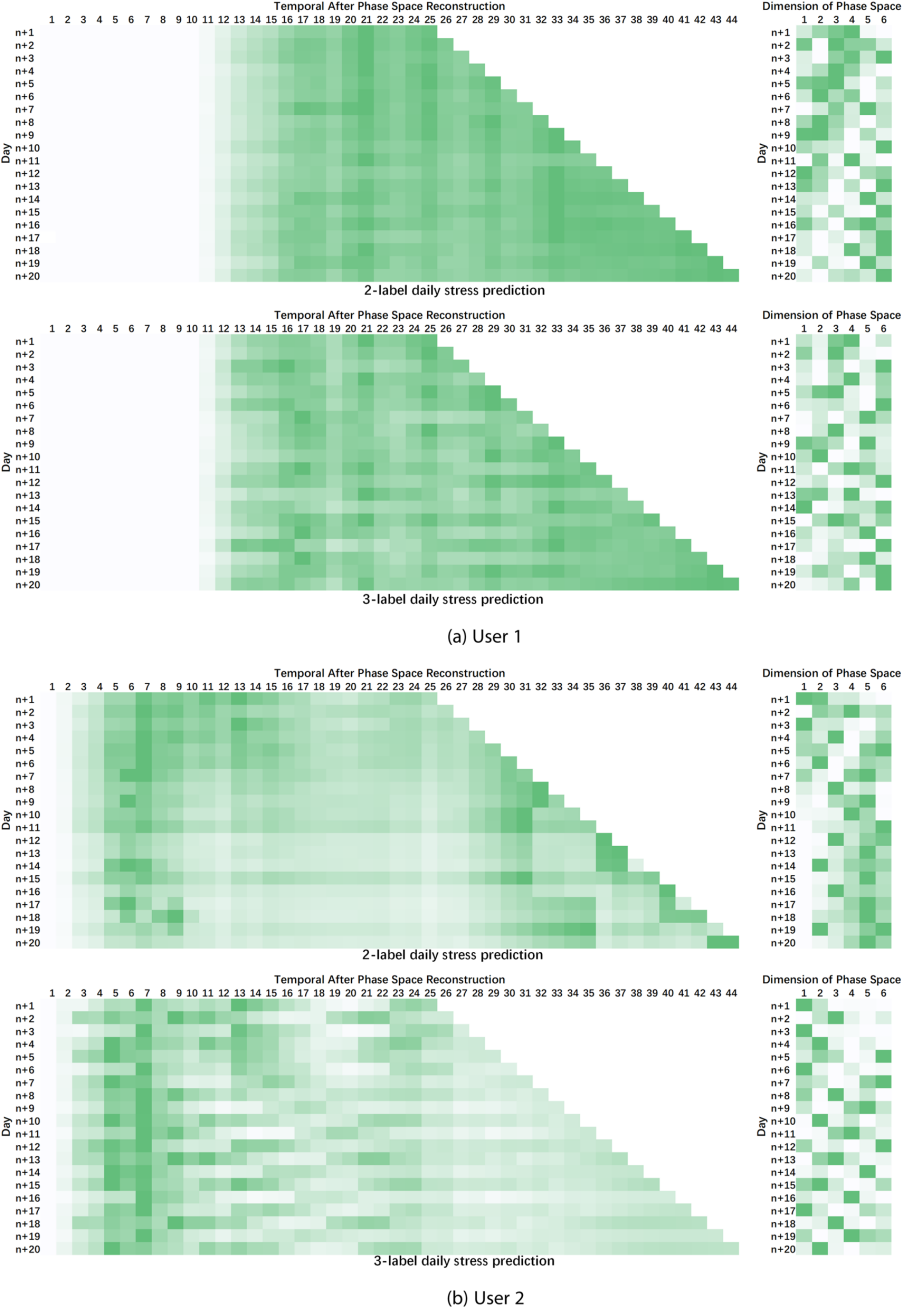
Temporal and dimension attention matrix of two users

*Personalized stress prediction.* In this study, we only consider using user’s historical stress levels to predict future stress states. However, users’ stress perception could be affected by various external factors and personal characteristics, such as personality traits, previous experiences, coping skills, social supports, etc. Figure 7 illustrates the dimension and temporal attention matrics of two users in the study. The darker the color, the greater the attention, signifying the importance for stress prediction. We can find that the attentions do not concentrate on certain specific parts. The attention of user 1 focuses more on the period closer to the predicted date, while the attention of user 2 focuses more on the earlier stage. To address the diversity, a personalized stress prediction framework, taking various stress related factors into account, is needed. These factors could be reflected as relevant parameters in the prediction model or built into the reconstructed high-dimensional personalized phase space.

*Providing interpretable stress prediction results.* A critical issue that deep learning faces is its interpretability. Deep neural networks are often considered as black-box models, meaning that their internal workings are hidden or difficult to explain. Recently, some studies have tried to address the interpretability of deep learning by perturbing the input to find out which parts of the input are important, calculating the importance of each input features to the network output, or visualizing a part of the parameters of the neural network to try to understand some structures inside the neural network [62]. In contrast, chaos theory attempts to describe the evolution process of nonlinear systems through attractors, fractals, self-similarity and other characteristics, and tries to describe the impact of small differences in inputs on output. Leveraging the characteristics of chaos theory, we might be able to provide interpretable stress prediction results.

*Adjusting the prediction model in an evolutionary fashion.* As shown in Figs. 3 and 6, we may find that the performance of our stress prediction model appears to be deteriorating with time. This may be because one’s stress response is a complex psychological phenomenon that is affected by many internal or external factors. The behaviors and characteristics of one’s stress may change over time. In order to make the model adapt to changes in people’s psychological states, in the future, we need to consider evolving the model over time through knowledge distillation, few-shot learning, etc. With these methods, the model could be flexibly adjusted according to new incoming data, helping people manage and reduce stress better.

## Conclusion

Chaos theory offers a unique perspective for understanding human mental states, while deep learning serves as a powerful tool for simulating the nonlinear state of human stress states. In this study, we validate the effectiveness of integrating deep learning and chaos theory for stress prediction. The experimental results on the publicly available Tesserae dataset show that the proposed method outperforms the pure deep learning method and Chaos method in both 2-label (unstressed/stressed) and 3-label (unstressed/weak stressed/heavy stressed) stress prediction, achieving 74.41% and 69.23% of accuracy in 2-label and 3-label stress prediction, which are over 2% and 8% more than those of the deep learning and the Chaos method, respectively. We are currently leveraging chaos theory to address the interpretability problem of deep learning so as to provide interpretable stress prediction results.

## Data Availability

The dataset we used in this paper is a publicly available Tesserae dataset.
